# Identification of a Novel Anti-cancer Protein, FIP-bbo, from *Botryobasidium botryosum* and Protein Structure Analysis using Molecular Dynamic Simulation

**DOI:** 10.1038/s41598-019-42104-1

**Published:** 2019-04-09

**Authors:** Ying Wang, Ying Nv Gao, Rui Bai, Hong Yu Chen, Ying Ying Wu, Jun Jun Shang, Da Peng Bao

**Affiliations:** 1 National Engineering Research Center of Edible Fungi, Key Laboratory of Applied Mycological Resources and Utilization, Ministry of Agriculture, Shanghai Key Laboratory of Agricultural Genetics and Breeding, Institute of Edible Fungi, Shanghai Academy of Agriculture Science, Shanghai, 201403 People’s Republic of China; 2 College of Food Science, Shanghai Ocean University, Shanghai, 201306 People’s Republic of China

## Abstract

Fungal immunoregulatory proteins (FIP) are effective small molecule proteins with broad-spectrum immunomodulatory and anti-cancer activities and can be potential agents for the development of clinical drugs and health food additives. In this study, a new member of FIP named FIP-bbo was obtained through *Botryobasidium botryosum* genome mining. FIP-bbo has the typical characteristics of FIP but is genetically distant from other FIPs. Recombinant FIP-bbo (rFIP-bbo) was produced in an optimized *E. coli* expression system, and the pure protein was isolated using a Ni-NTA column. Antineoplastic experiments suggested that FIP-bbo is similar to LZ-8 in inhibiting various cancer cells (Hela, Spac-1, and A549) at lower concentrations, but it is not as potent as LZ-8. The molecular mechanism by which FIP-bbo, FIP-fve, and LZ-8 are cytotoxic to cancer cells has been discussed based on molecular dynamics simulation. Point mutations that may improve the thermal stability of FIP-fve and FIP-bbo were predicted. These results not only present a new candidate protein for the development of anticancer adjuvants, but also provide an approach for designing FIPs with high anticancer activity.

## Introduction

Fungal immunoregulatory proteins (FIP) are small molecules obtained from various fungi that have similar amino acid compositions and protein structures^1^. FIPs possess a certain degree of homology with the heavy chain variable region of human and mouse immunoglobulins. Thus, FIP may be related to the immunoglobulin superfamily^1^. A comparison of the crystal structures of FIP isolated from *Flammulina velutipes* (PDB ID:1OSY)^2^, *Ganoderma lucidum* (PDB ID:3F3H)^3^, and *G. microsporum* (PDB ID:3KCW) showed that they are non-covalently linked homo-dimers, and each monomer comprises an N-terminal α-helix followed by a FNIII fold.

FIP have various immunomodulatory activities and are important to researchers. Several experiments showed that FIP is a potent T cell activator that enhances the immune response by modulating the production of important cytokines and molecular factors (IL-2, IFN-γ, IL-1β, TNF-α etc.) in several vertebrate model systems^4^. The ability of these cytokines to produce a tumour suppressing environment indirectly proves that FIP regulates the immune system and has a cytotoxic effect on tumour cells^5^. Moreover, evidence show that FIP can directly inhibit cancer proliferation and migration by inducing apoptosis in tumour cells^6^.

Despite being highly conserved with respect to its sequence and structure, FIP from different sources may possess different activity of fighting against cancer. Previous studies demonstrated that recombinant LZ-8 (rLZ-8), FIP from *G. lucidum*, inhibits A549 lung cancer cell progression by promoting EGFR degradation^7^. The FIP-fve, FIP from *F. velutipes*, inhibits A549 lung cancer cells migration and invasion by decreasing RacGAP1 expression and Rac1 activity^8^. Although both rLZ-8 and FIP-fve prevented lung cancer cell proliferation via the increase G1 arrest, their effect strengths are different. The anti-cancer effect of LZ-8 is stronger than that of the FIP-fve^8–10^.

Only a few fungi contain FIP and considering the drawbacks related to fungal samples, it takes a lot of effort to identify new FIPs from the fruiting body or mycelium through isolation or cloning using a universal primer. However, the progress and affordability of genomic sequencing technology is transforming the way FIPs are discovered. Even without fungal samples, the gene sequence of FIPs such as FIP-nha^11^, FIP-ppl^12^, FIP-sch2^13^, FIP-Lrh^14^, and FIP-dsq2^15^ can be obtained by analysing the enormous genomic information available online.

Here, FIP-bbo, a novel FIP from *Botryobasidium botryosum*, was obtained by performing a sequence similarity search in a rapidly evolving genome database. FIP-bbo is similar to LZ-8 in that it inhibits cancer cells (Hela, Spac-1, and A549) at lower concentrations; however, its activity was not as high as LZ-8. The FIP-bbo molecular model was constructed by homology modelling using LZ-8, GIM, and FIP-fve crystal structures as templates. The molecular mechanism by which FIP-bbo, FIP-fve, and LZ-8 induce cytotoxicity in cancer cells has been discussed using molecular dynamics simulation.

## Results

### A novel FIP: FIP-bbo

The 336 base pairs (bp) FIP-bbo comprises 112 amino acids and has a molecular weight of 12.42 kDa. FIP-bbo with a pI value of 6.54 is an alkaline protein with 11 strongly basic (+) amino acids (Arg, Lys) and 11 strongly acidic (−) amino acids (Asp, Glu). FIP-bbo is a stable protein with an instability index (II) of 6.29 and lacks a signal peptide and transmembrane helices. As described above, FIP-bbo possess the typical characteristics of FIP.

**Figure 1:**
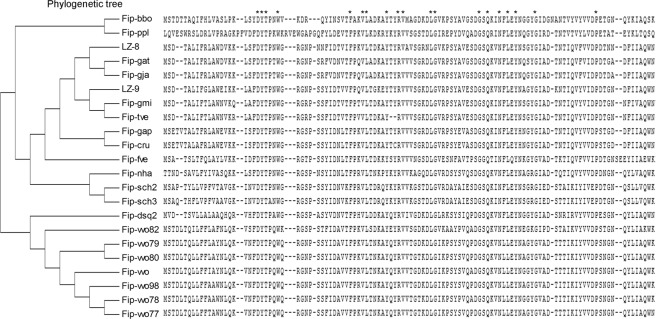
Phylogenetic tree and the sequence alignment of FIP.

The dendrogram based on the FIP amino acids analysis showed that FIP is mainly divided into three branches (Fig. 1). The FIP from *Ganoderma* spp. (LZ-8, FIP-gat, FIP-gja, LZ-9, FIP-gmi, FIP-gts, and FIP-gap), additionally, FIP-cru, FIP-tve, FIP-fve, FIP-nha, FIP-sch2, and FIP-sch3 were derived from the same evolutionary process. The FIP from *Volvaria volvacea* (FIP-vvo82, FIP-vvo79, FIP-vvo80, FIP-vvo, FIP-vvo98, FIP-vvo78, and FIP-vvo77) and FIP-dsq2 were clustered into the second lineage. FIP-ppl and FIP-bbo alone formed a separate lineage. Sequence alignment analysis indicated that the central region is more conserved than the N-terminal and C-terminal region.

**Figure 2:**
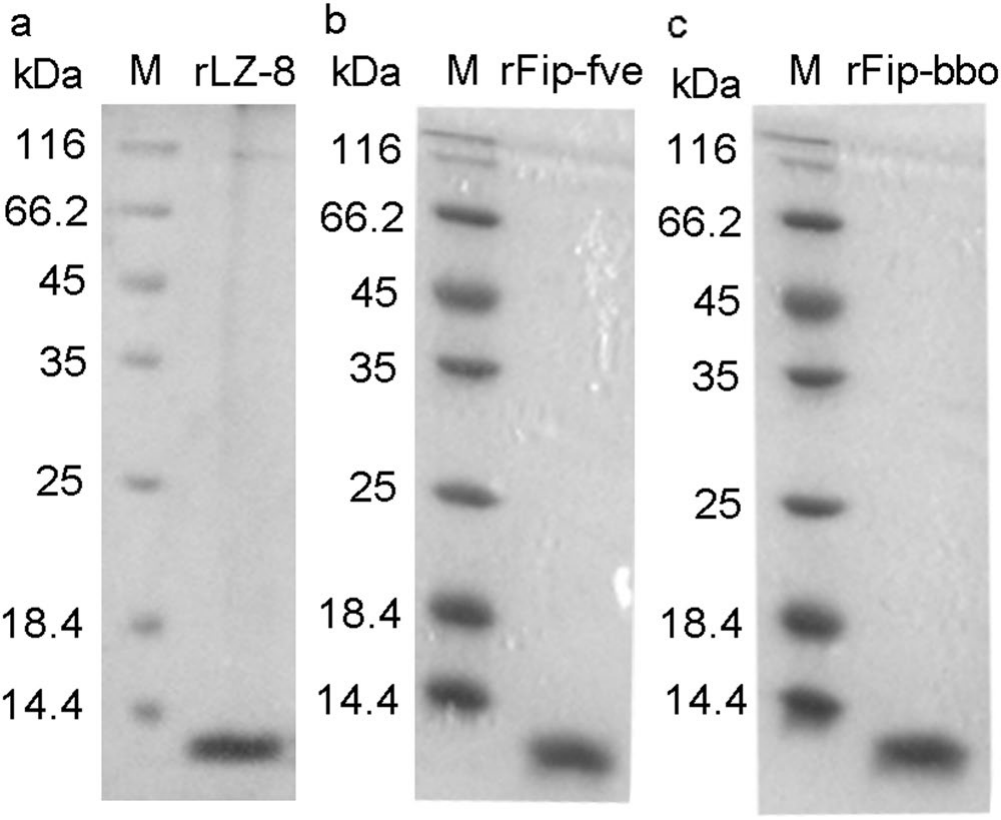
SDS-PAGE analysis of purified recombinant FIP expressed by *E. coli*. The cropped images originate from one gel. The full-length gel is presented in Supplementary Fig. S4.

The DNA fragments encoding LZ-8, FIP-fve, and FIP-bbo were cloned and the FIP were expressed as a SUMO fusion protein in *E. coli*. The expression conditions (induction temperature and concentration of the inducer) were optimized (Fig. S1). Results obtained using SDS-PAGE confirmed that the fusion protein was more soluble at an induction temperature of 37 °C (Fig. S2). Therefore, the amplified expression was finally induced with 1.0 mM IPTG at 37 °C, and the SUMO-tag was removed after purification using Ni-NTA to obtain the target protein (Fig. S3). Finally, the LPS was removed. A band of the three FIP was observed near 13 kDa using a SDS-PAGE, as suggested by the theoretical calculations (Fig. 2).

### Effect of rFIP-bbo on inhibiting the proliferation of Hela, Spca-1 and A549 cells

**Figure 3:**
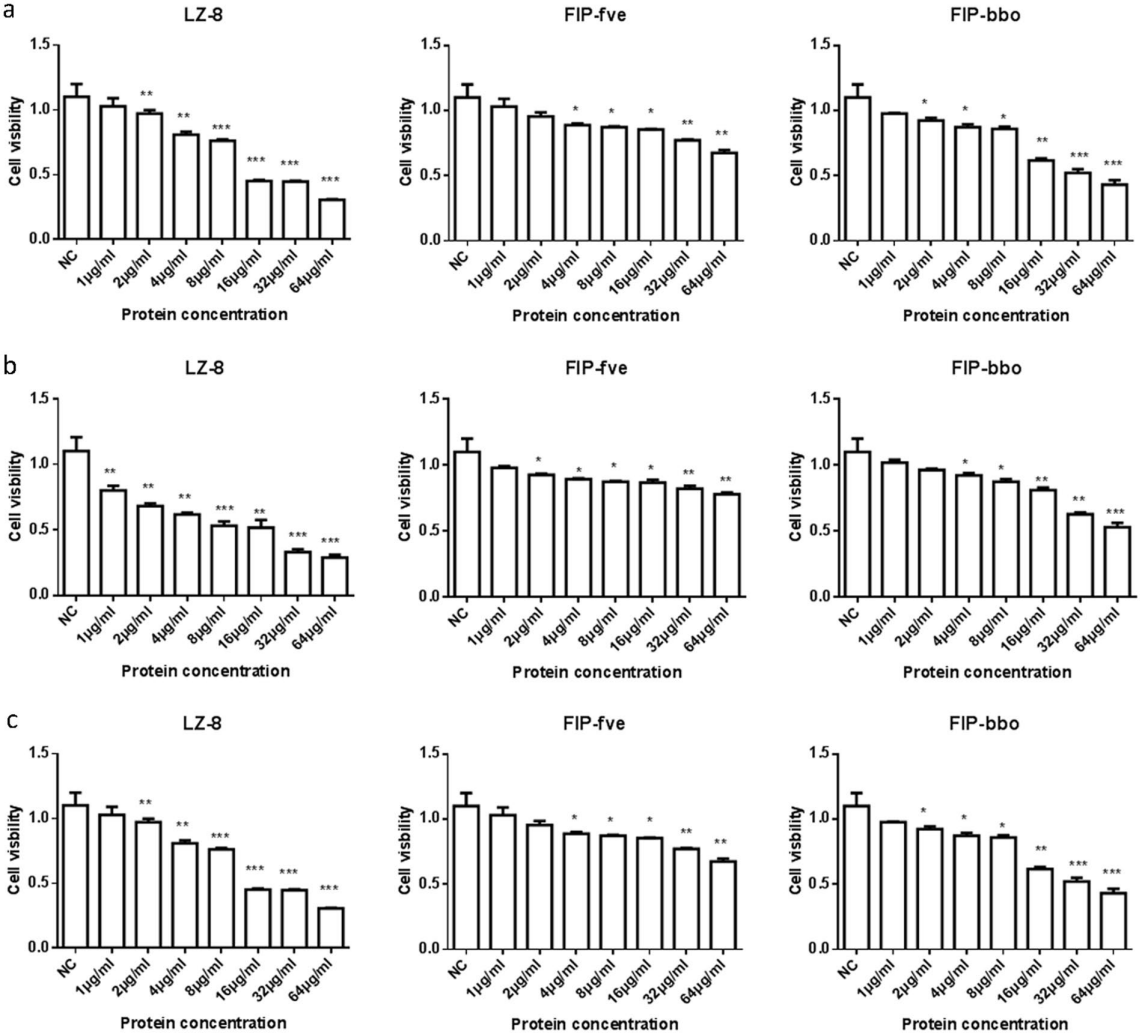
Proliferation inhibition by rFIP on Hela (**a**), Spac-1 (**b**) and A549 (**c**) cells. These cells were treated with 1, 2, 4, 8, 16, 32, and 64 μg/mL rFIPs for 16 h, compared with negative control. *p < 0.05, 0.05 > **p > 0.001, 0.001 > ***p > 0.0001, ****p < 0.0001. Each experiment was repeated at least thrice.

Former studies have shown that FIP has potential effects against tumours, such as rLZ-8 and rFIP-fve. Therefore, the antineoplastic effect of rLZ-8, rFIP-fve and rFIP-bbo was studied on Hela, Spca-1 and A549 cells at various concentrations (1, 2, 4, 8, 16, 32 and 64 μg/ml) *in vitro* by MTT assay. As shown as Fig. 3, the rFIP significantly suppressed proliferation of Hela, Spca-1 and A549 cells. However, rFIP-fve was not particularly sensitive to proliferation inhibition of cancer cells with respect to rLZ-8 and rFIP-bbo. LZ-8 had the best inhibitory activity.

### Effect of rFIP-bbo on stimulating apoptosis in Hela, Spca-1, and A549 cells

**Figure 4:**
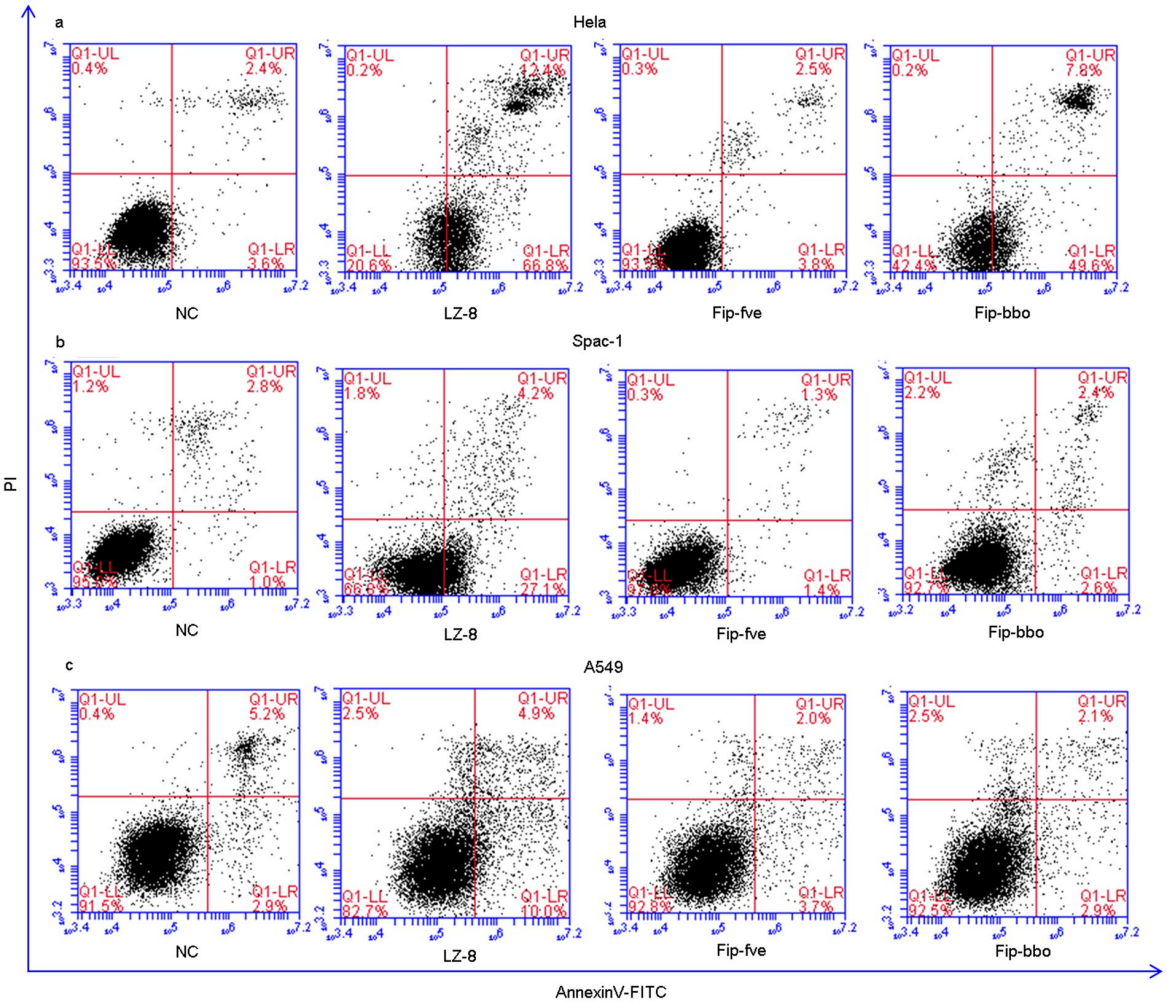
Apoptosis induced by FIP. 8 μg/mL of rLZ–8, rFIP-fve and rFIP-bbo were added into the Hela, Spca-1 and A549 cells, incubated for 24 h and analysed using a flow cytometer to detect the rate of apoptosis.

The apoptosis of Hela, Spca-1, and A549 cells *in vitro* by rLZ-8, rFIP-fve and rFIP-bbo was detected by flow cytometry (FCM) (Fig. 4). For example, by treatment of Hela with 8 μg/ml of rLZ-8, rFIP-fve, and rFIP-bbo for 24 h, the rate of inhibition of the proliferation of Hela cells was found to be 79.2%, 6.3% and 57.4%, respectively. Our results were consistent with previous studies in which rLZ-8 had significant antitumor activity, but rFIP-fve had no significant effect at a lower concentration.

**Figure 5:**
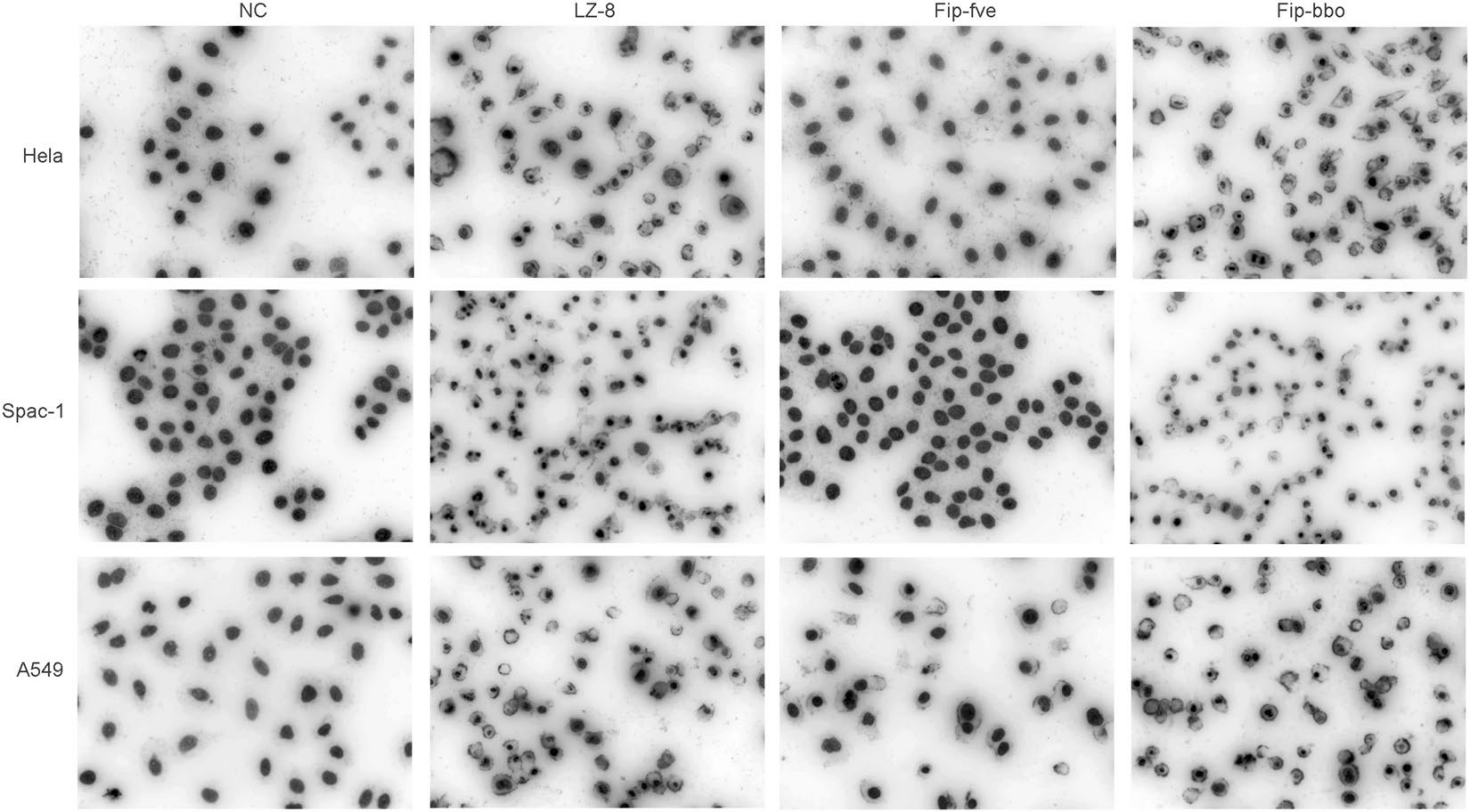
Cytotoxicity assay of FIP. Hela, Spca-1 and A549 cells were treated with 8 μg/mL of rLZ–8, rFIP-fve and rFIP-bbo and apoptosis was detected using Hoechst Staining Kit.

From the result obtained using the Hoechst Staining Kit, rFIP-fve (8 μg/ml) had no remarkable inducing effect on apoptosis of Hela, Spca-1, and A549 cells (Fig. 5). However, with the treatment of 8 μg/ml rLZ-8 or rFIP-bbo, the nucleus of the cells showed dense thick dye or chopped dense dye. This indicated that rLZ-8 and rFIP-bbo condense the chromatin. This phenomenon was similar to apoptosis.

### Effect of rFIP-bbo on inhibiting the migration of Hela, Spca-1, and A549 cells

**Figure 6:**
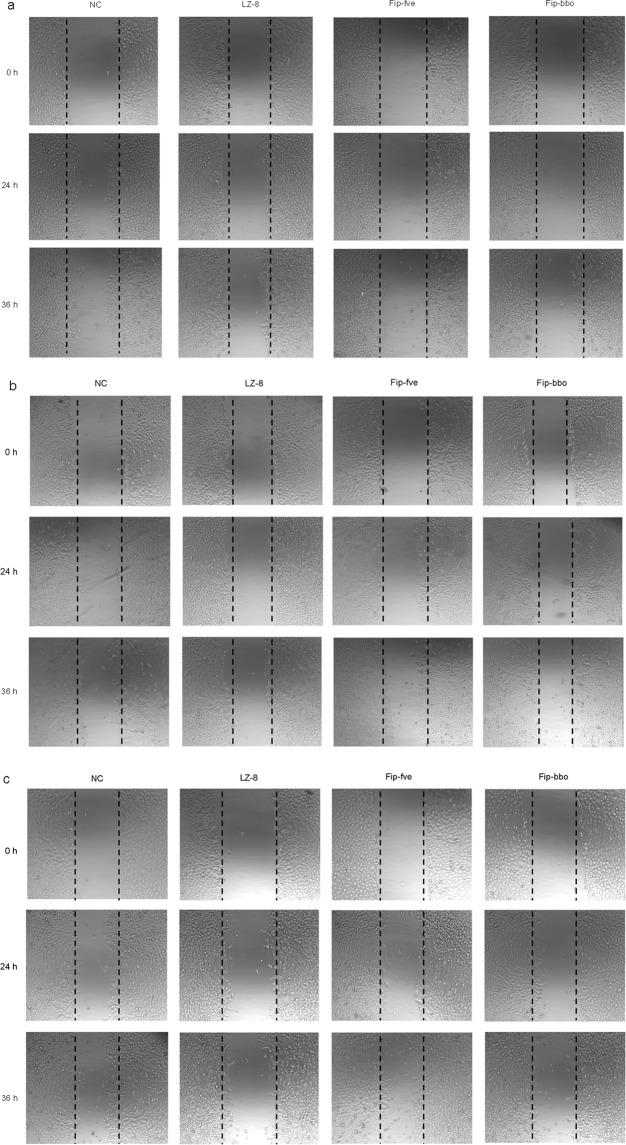
Effect of FIP on migration of Hela, Spca-1 and A549 cells by wound healing assay. Hela Spca-1 and A549 cells were treated with 8 μg/mL rLZ-8, rFIP-fve, and rFIP-bbo for 36 h. PBS was used as a negative control (NC). Cell migration was observed by microscopy.

Cancer metastasis is the leading cause of death in cancer patients. Early studies have shown that LZ-8 inhibited the migration of some cancer cells. Using rLZ-8 as a positive control, we observed the inhibitory effect of rFIP-bbo on migration of Hela, Spca-1, and A549 cancer cells. A concentration of 8 μg/mL of rLZ-8/rFIP-fve/rFIP-bbo was added to cancer cells of culture, and at 0 h, 24 h, and 36 h their migration to the streaked area was observed, respectively. As shown in Fig. 6, the cancer cells that were treated with PBS as the negative control (NC) on the left and right side merged after treatment for 36 h. Cells that were treated with rLZ-8 and rFIP-bbo demonstrated decreased wound closure activity. However, rFIP-fve exhibited a stronger wound closure activity that was similar to that of the NC. The results proved that rFIP-bbo suppressed Hela, Spca-1 and A549 cell migration to the denuded zone, and the migration inhibition effect of rFIP-bbo was slightly lower than that of rLZ-8, but higher than that of rFIP-fve.

### Molecular dynamics simulation of FIP

**Figure 7:**
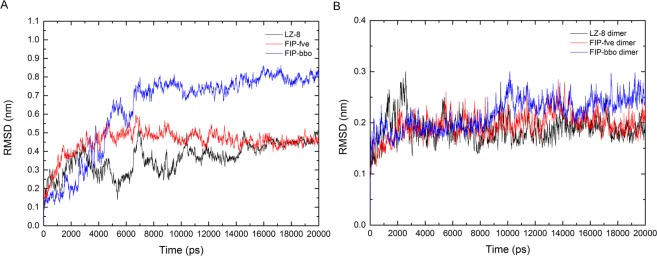
Root-mean-square deviations (RMSD) for all the atoms of FIP monomers (**A**) and dimers (**B**) from the initial structure.

We used molecular modelling techniques to study the static and dynamic structure of FIP, and to determine their varied activities. Referring to the crystal structure of LZ-8 and FIP-fve, the 3D structure of rFIP-bbo protein was constructed using MODELLER software, and the model was optimized by using the CHARMM27 force field for energy minimization. The root mean square deviation (RMSD) is considered to be an indicator of structural stability. RMSD analysis showed that each FIP monomer simulation system converges after 7 ns (Fig. 7A), LZ-8 dimer and FIP-fve dimer simulation system converges after 3 ns, FIP-bbo dimer simulation system converges after 10 ns (Fig. 7B), and structural dynamics and thermodynamic analysis can be performed, which on closer examination revealed that in FIP monomer and dimer simulation systems, the LZ-8 had the lowest RMSD value. Compared the RMSD plots of the monomers and dimer systems revealed that the formation of dimer further stabilized the FIP’s structures.

**Figure 8:**
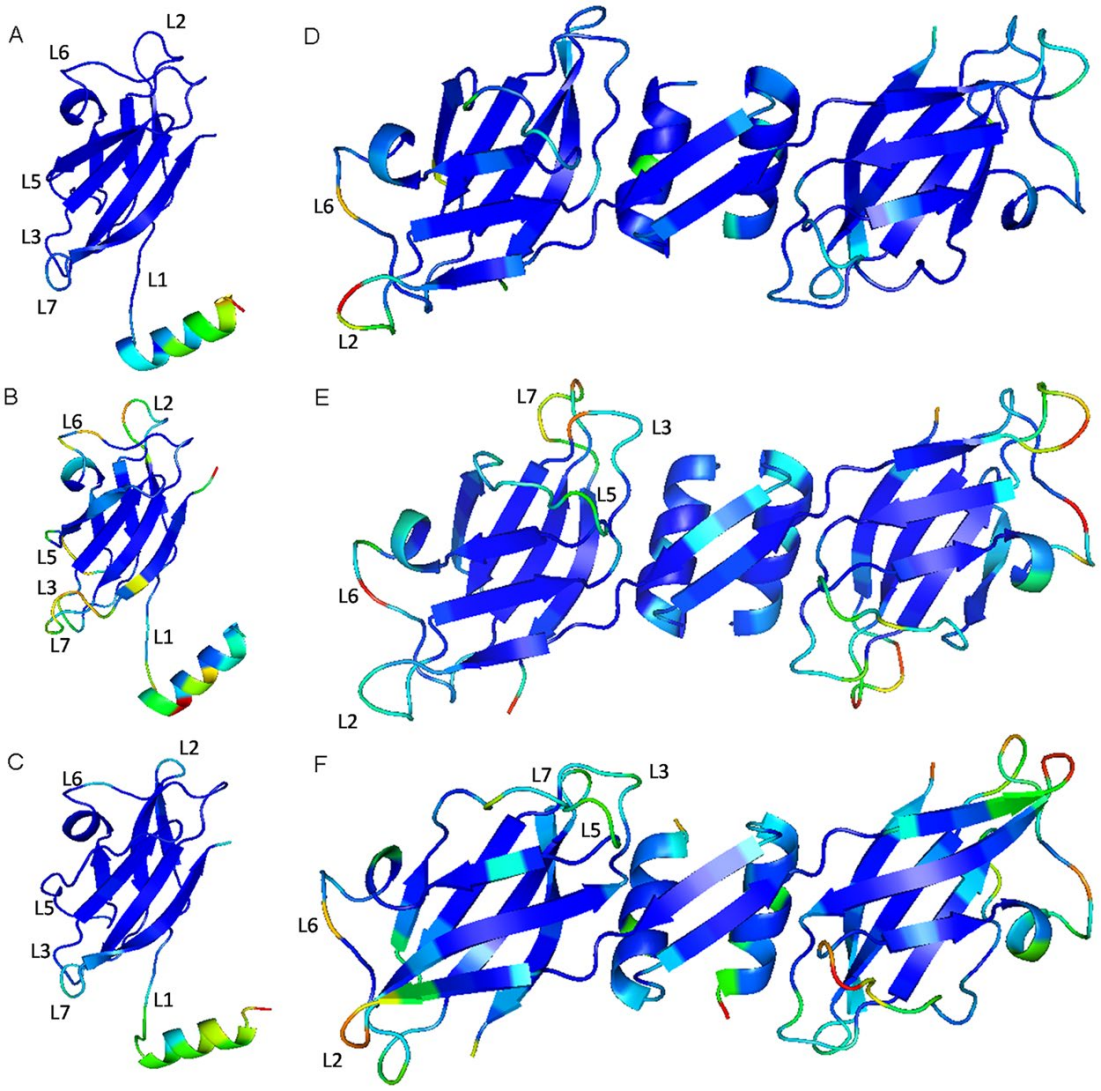
3D structures of FIP ((**A**) LZ-8 monomer, (**B**) FIP-fve monomer, (**C**) FIP-bbo monomer, (**D**) LZ-8 dimer, (**E**) FIP-fve dimer and (**F**) FIP-bbo dimer) with B-factor. The B-factor value of an amino acid is obtained by taking an average of all a B-factor atoms that make up the amino acid. The B-factor values are represented by red, yellow, green, and blue in descending order.

Temperature factor, also known as B-factor, is an ambiguity used to describe the state of atomic conformation in a structure. It reflects the conformational state of protein molecules in the structure. The higher the B-factor, the greater the ambiguity, and the more unstable or flexible the conformation of the corresponding parts^16^. Most parts of the FIP structure were very stable (blue), and a few parts were flexible (green, yellow or red) (Fig. 8). In the FIP monomer MD simulation systems, all the parts except the N-terminal α-helix of LZ-8 were stable. The FIP-bbo structural stability was similar to LZ-8, but the flexibility of the N-terminal α-helix was higher than LZ-8. However, in addition to the N-terminal α-helix, there are 6 loop structures (L1, L2, L3, L5, L6 and L7) of FIP-fve with flexibility, which proves that the dynamic change of FIP-fve in solution is different from that of LZ-8 and FIP-bbo. Sorting the B-factor values of the amino acids that make up the FIP-fve and FIP-bbo, and then replacing the amino acids that have high B-factor values with amino acids that have low B-factor values of LZ-8 can improve the thermal stability of the protein. The specific scheme is to combine B-Factor with multisequence alignment^16^. Therefore, point mutations in FIP-bbo (R31N, Q32N and E101D) and in FIP-fve (T29N, K47A, T68E, P69S, S70D, K82S, S104N and E105N) would increase the stability of FIP-bbo and FIP-fve. As shown as Fig. 8, when monomers bound to each to form homo-dimer, the stability of the various parts of FIP had changed. The N-terminal α-helix of FIP became stable, but the outer loop (L2 and L6) of the LZ-8 dimer became flexible. And the flexibility of FIP-bbo dimer’s loops (L2, L3, L5, L6 and L7) was between LZ-8 and FIP-fve.

### Molecular mechanism of difference in anticancer activity of FIP

**Figure 9:**
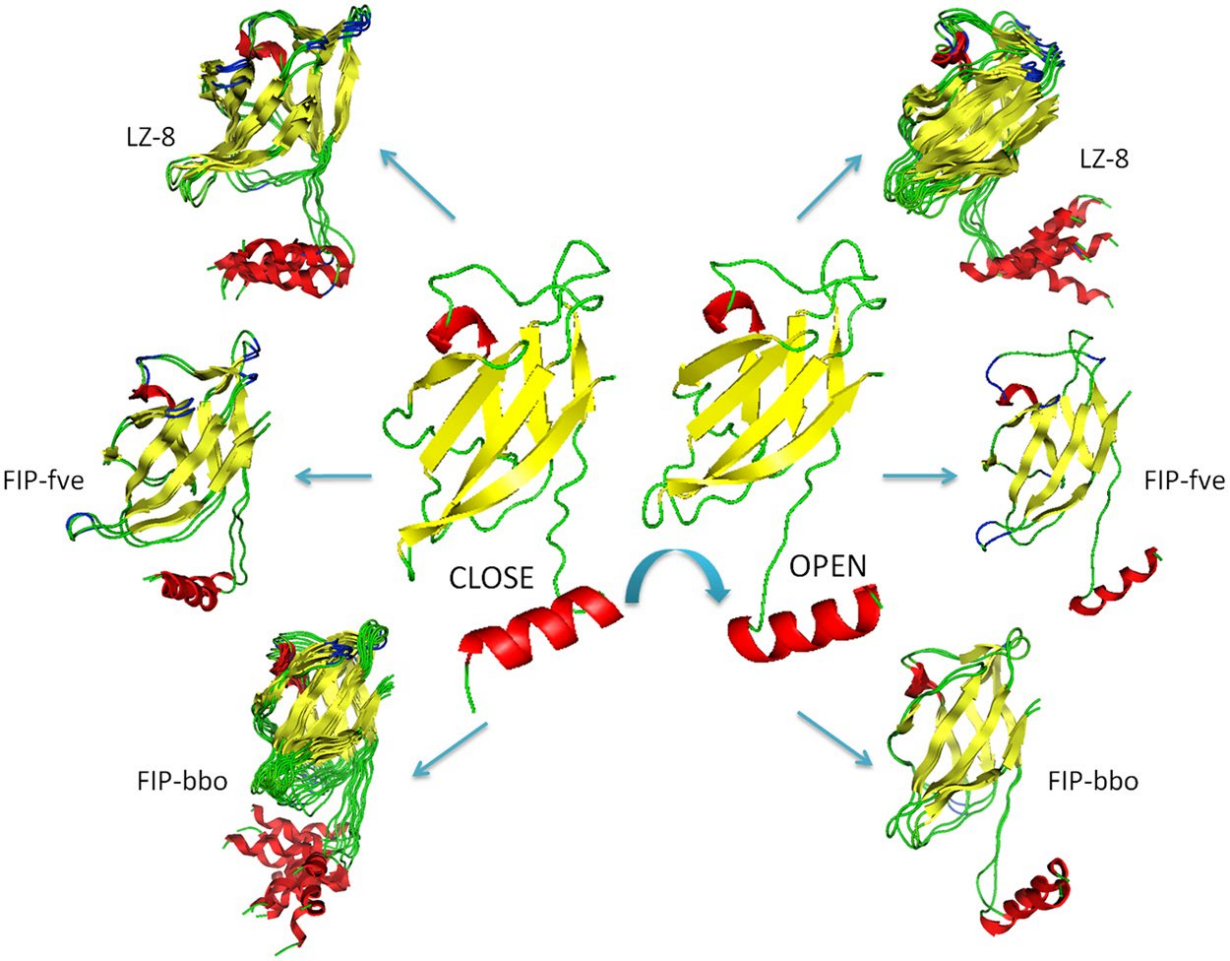
The structures of the three simulation systems by cluster analysis. The conformational changes of LZ-8, FIP-fve and FIP-bbo from cluster analysis. Their formation of homo-dimers is decided by the directions of N-terminal α-helix. If N-terminal α-helix turns to the left, the FIP becomes the closed structure, and when it turns to the right, it becomes the opened structure.

Through detailed structural analysis, 2001 protein conformations in the simulated trajectory of FIP are classified by the cluster analysis tool. This, combined with the results of protein docking analysis of the average protein conformation of each FIP cluster, showed that all protein conformations in the simulated trajectory were divided into two categories, one was the closed conformation, which could not form a homo-dimer similar to the crystal structure of FIP-fve/LZ-8, and the other was the opened conformation (Fig. 9). In the simulated trajectory of FIP-fve, all protein conformations were in 3 clusters, with the first cluster (1900 protein conformations) being predominant. The average protein conformation of cluster 1 and cluster 2 was the closed conformation. The average conformation of the second cluster with only 78 conformations accounting for 3.9% of the simulation time was opened structure. In LZ-8, all protein conformations were in 12 clusters, the average conformation of 5 clusters (cluster 1, 3, 4, 9 and 11) was the closed conformation, and the average conformation in the remaining clusters is the opened conformation. FIP-bbo trajectories of all protein conformations were in 14 clusters, the average conformation of 2 clusters (cluster 2 and 13) was the opened conformation, and the average conformation in the rest clusters is the closed conformation.

**Figure 10:**
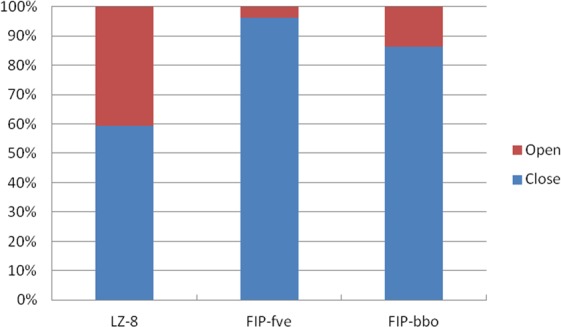
The proportion of the open structure in the molecular dynamics trajectory.

On the basis of cluster analysis, the statistical proportion of the opened structure in the molecular dynamics trajectory is shown in Fig. 10. In the simulated trajectory of LZ-8, FIP-fve and FIP-bbo, the number of opened conformation accounts for 40.6%, 3.9% and 13.7% of the all protein conformation, respectively. When the FIP is in an opened state, the home-dimer may be formed. If it is considered that greater the number of protein conformations that can form homo-dimers, easier is its conversion to homo-dimers, LZ-8 was most likely to form a homo-dimer, followed by FIP-bbo, and FIP-fve. This is consistent with above FIP antitumor activity results.

**Figure 11:**
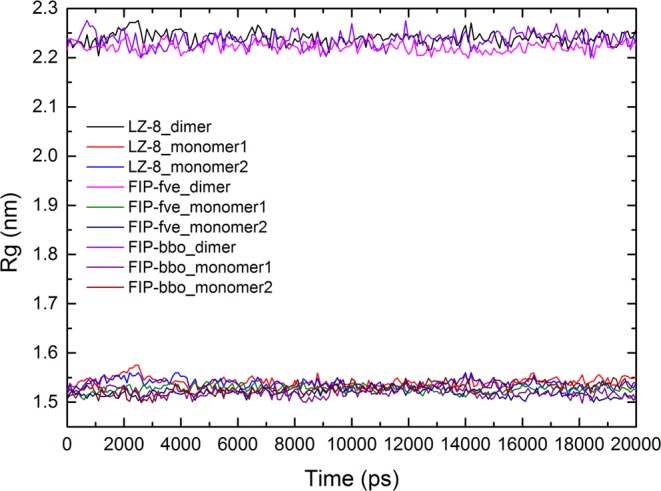
Radius of gyrations for all the C*α* atoms of FIP dimers during the molecular dynamics simulation.

In addition to the molecular dynamics simulation of FIP monomers, we also performed molecular dynamics simulation analysis of homo-dimers. And MM-PBSA calculations were applied for calculating binding free energies and its components of the FIP homo-dimer. The calculated results are listed in Table 1. Analysis of the binding free energy of each FIP monomer revealed that the dimer formed by FIP-fve was most stable. However, the ability to form homo-dimer between LZ-8/FIP-bbo monomers was weak. Further analysis revealed that the electrostatic solvation free energy between FIP-fve monomers were significantly higher than LZ-8 and FIP-bbo. Meanwhile, the analysis of radius of gyration (Rg) of each FIP revealed that the protein volume of FIP-fve dimer is smaller than LZ-8 and FIP-bbo, and stabilized as a compact (folded) form in a stable state (Fig. 11).

**Table 1:** Binding energies between monomers in the homo-dimers of LZ-8, FIP-fve and FIP-bbo (kJ/mol).

	LZ-8 dimer	FIP-fve dimer	FIP-bbo dimer
ΔG_elec_	−1094.261	−1447.881	−1025.375
ΔG_vdw_	−601.803	−658.872	−547.259
ΔG_sol___polar_	857.280	910.807	797.884
ΔG_sol_nonpolar_	−71.763	−75.504	−66.059
ΔG_polar_^a^	−198.661	−537.074	−227.491
ΔG_nonpolar_^b^	−673.566	−734.376	−613.318
ΔG_bind_	−910.929 ± 16.241	−1271.901 ± 19.007	−839.976 ± 17.797

^a^ΔG_polar_ = ΔG_elec_ + ΔG_sol___polar_.

^b^ΔG_nonpolar_ = ΔG_vdw_ + ΔG_sol_nonpolar_.

## Discussion

Numerous studies have shown that FIP possess broad spectrum immunomodulatory and anticancer activities, suggesting that they may be promising candidates in clinical disease therapies or for treatment as adjuvant agents^4,17^. FIP can be orally consumed and have high stability when exposed to denaturing conditions^18,19^. These characteristics make FIP a potential candidate to be used as an additive in the health food industry. In this study, a novel FIP, FIP-bbo, with anticancer activity was found from *B. botryosum* through genome mining. *B. botryosum* is a basidiomycete species that produces thin, resupinate, greyish to yellowish basidiocarps on wood debris and characteristic navicular basidiospores. But the ecology and nutritional strategy of this species, as well as other Botryobasidium spp., is not well known. Only some species have been collected from brown-rotted wood^20^. Therefore, to produce sufficient quantities of FIP-bbo for our analysis, a recombinant expression strain was developed.

Experiments on cell proliferation, apoptosis and migration in cancer cells such as Hela, Spca-1 and A549 revealed that LZ-8, FIP-fve and FIP-bbo have anticancer activities, but their effective concentrations are different. At lower concentrations, both LZ-8 and FIP-bbo have potency in inhibiting cancer cells, but LZ-8 is more potent. FIP-fve has anticancer activities at higher concentrations, which is consistent with previous studies. These differences are closely related to the high-level structure of the FIP protein.

Research on elucidating the structure of the FIP has already made some progress. For example, the crystal structures of LZ-8 and FIP-fve have been resolved, and it has been found that the difficulty in the formation of homodimers and the stability of these dimers directly affect the activity of FIP^21–23^. Thus, this experiment used molecular dynamics simulation to study the dynamic changes of FIP in solution to explore and understand the molecular model of its anticancer activity. Through monomer and dimer simulations, the results obtained from stability experiments showed that LZ-8 was most stable, followed by FIP-fve and FIP-bbo (Figs 7 and 8). Through the B-factor, we found key amino acids which caused the FIP-fve and FIP-bbo proteins to destabilize. Point mutations R31N, Q32N and E101D in FIP-bbo and T29N, K47A, T68E, P69S, S70D, K82S, S104N and E105N in FIP-fve were done to improve their stability.

Through molecular dynamics analysis of LZ-8, FIP-fve and FIP-bbo, it was found that the probability of forming homo-dimers varied, and their stabilities were different. We inferred that the protein structure of LZ-8 could be in a stable opened state in which homo-dimer would form over a relatively long period of time (40.6% of the entire simulation). FIP-fve was in this state for a very short time (3.9% of the entire simulation), which was only one-tenth the amount of time that LZ-8 was in this state. This provides a theoretical basis for FIP-fve to be active only at higher concentrations. Increasing its concentration could increase the probability of forming a homo-dimer. On the contrary, LZ-8 has more time to form a homodimer, so LZ-8 can produce activity at lower concentrations. The time with respect to FIP-bbo (13.7% of the entire simulation) is lesser than LZ-8 but greater than FIP-fve. Therefore, the antitumor activity of FIP-bbo is similar to, but slightly lower than that of LZ-8. Although FIP-fve is less likely to form homodimers, it is very stable once dimers are formed. This may be the reason why the crystal structure of FIP-fve is a homodimer.

## Conclusion

FIP-bbo is a novel member of the FIP family mined from *B. botryosum*. The physicochemical characterization of FIP-bbo is similar to the characteristics observed in the FIP. However, the FIP-bbo evolves independently in the two branches of FIP. Anti-proliferation, pro-apoptosis, and inhibiting migration experiments on Hela, Spca-1 and A549 showed that rFIP-bbo has anticancer activity. The anticancer activity of the rFIP-bbo lies between that of rLZ-8 and rFIP-fve. Molecular dynamics simulation analysis revealed the molecular mechanism observed of the anticancer activity shown by LZ-8, FIP-fve, and FIP-bbo through thermodynamics and molecular structures. The point mutations (R31N, Q32N and E101D in FIP-bbo and T29N, K47A, T68E, P69S, S70D, K82S, S104N and E105N in FIP-fve) may have improved the thermal stability. The study not only identifies a novel candidate protein for the development of an anticancer adjuvant, but also provides the basis for designing an FIP with high anticancer activity.

## Materials and Methods

### Source of data and bioinformatics analysis

The genome sequence of *B. botryosum* (GenBank: GCA_000697705.1) and amino acid sequences of LZ-8 (GenBank: ACD44335.1) and FIP-fve (GenBank: P80412.1) were downloaded from NCBI (https://www.ncbi.nlm.nih.gov/). The protein structures of LZ-8 (PDBID: 3F3H) and FIP-fve (PDBID: 1OSY) were obtained from the Protein Data Bank (PDB). A BLAST search was performed using the BLAST software. The MEGA7 package^24^ was used for reconstruction of a phylogenetic tree, and neighbour-joining trees were inferred by the program.

### Homology modelling of FIP-bbo

Using a protein modelling software, MODELLER^25^, homology modelling of FIP-bbo was performed with the crystal structure of FIP using *G. lucidum* (PDBID: 3F3H), *G. microsporum* (PDB ID:3KCW), and *F. velutipes* (PDBID: 1OSY) as templates. Using MODELER, 50 models were produced and ranked by the value of the objective function and the score of discrete optimized potential energy. The model with the highest score was selected as the three-dimensional (3D) structure of FIP-bbo and optimized by a 1000-step energy minimization process using the steepest descent method. The quality of the FIP-bbo 3D structures was evaluated using PROCHECK^26^.

### Molecular dynamics simulation

The monomers and dimers of LZ-8, FIP-fve and FIP-bbo were simulated for 20 nanoseconds (ns), respectively. The molecular dynamics simulations were performed using GROMACS v5.0.2 simulation package^27^, with CHARMM27^28^ force field parameters and periodic boundary conditions. The initial models were solvated in 7 × 7 × 7 nm boxes with SPC water and a space of 10 Å around the solute. Sodium and chloride ions were randomly placed to neutralize and maintain the systems’ ionic concentration of 153 mmol/L. Before molecular dynamics simulation, the minimization with the steepest descent algorithms of 50000 steeps and two phases equilibration (NVT ensemble and NPT ensemble) was conducted. Finally, a 20 ns molecular dynamics simulation was performed at 310 K on the whole system. The time of the simulation was set at 2 fs, and the coordinates were documented for analysis every 1 ps. Post-processing the data was analysed using standard GROMACS tools and customized Perl scripts. The visualization of the refined model was performed by PyMOL software^29^.

### Protein-protein docking

The docking between the homo-monomers of LZ-8, FIP-bbo and FIP-fve were performed by ZDOCK^30^ and RDOCK^31^. ZDOCK searches a space of exclusively symmetric multimers for the best structure using a grid-based Fast Fourier Transform (FFT) approach. Following which, the structural minimization and rescoring of initial-stage docking models was performed by RDOCK.

### MM-PBSA calculations

To study the interaction between the FIP monomers in the FIP dimer, the molecular mechanics Poisson Boltzmann surface area (MM/PBSA) analysis was performed using the g_mmpbsa tool of GROMACS^32–34^. The MMPBSA method for calculation of the binding energy in general can be summarized as:

1 $${{\rm{\Delta }}{\rm{G}}}_{{\rm{bind}}}={{\rm{G}}}_{{\rm{dimer}}}-{{\rm{G}}}_{{\rm{monomer}}1}-{{\rm{G}}}_{{\rm{monomer}}2}$$

The G_dimer_, G_monomer1_ and G_monomer2_ are the free energies of FIP dimer and FIP monomers in solvent, respectively. The free energies were estimated by:

2 $${\rm{G}}={{\rm{E}}}_{{\rm{MM}}}+{{\rm{G}}}_{{\rm{sol}}}$$

3 $${{\rm{E}}}_{{\rm{MM}}}={{\rm{E}}}_{{\rm{int}}}+{{\rm{E}}}_{{\rm{ele}}}+{{\rm{E}}}_{{\rm{vdw}}}$$

4 $${{\rm{G}}}_{{\rm{sol}}}={{\rm{G}}}_{{\rm{polar}}}+{{\rm{G}}}_{{\rm{nonpolar}}}$$

E_MM_ stands for the gas phrase energy, which consists of the electrostatic energy (E_ele_), the van der Waals interaction energy (E_vdw_) and the sum of the internal interaction (E_int_). The solvation free energy (G_sol_) is made up of the electrostatic solvation free energy (G_polar_) and the aplolar salvation free energy (G_nonpolar_). Poisson–Boltzmann approaches can be used to calculate G_polar_ and G_nonpolar_ is estimated from the solvent-accessible surface area (SASA).

### Expression and purification of recombinant FIP from *E. coli*

The genes encoding FIP-bbo (KDQ10166), LZ-8 (P14945), and FIP-fve (P80412) were optimized for *E. coli* and were synthesized by General Biosystems Limited. The expression vector used was PCreat-SII (N-terminal carries His-tag and SUMO-tag) to produce the recombinant FIP (rFIP-bbo, rFIP-fve and rLZ-8), and BamHI and XHoI were the enzyme cleavage sites. The recombinant plasmid was transformed into *E. coli* BL21 (DE3), and positive colonies were isolated. Its expression was induced by isopropyl-β-D-thiogalactoside (IPTG) and was visualized using SDS-PAGE. The His-tag and lipopolysaccharides (LPS) were removed.

### Cell proliferation inhibitory assay

Human cancer cell lines including cervical carcinoma cell Hela, lung adenocarcinoma cells A549, and Spca-1 were obtained from the Shanghai Institute of Biochemistry and Cell Biology and cultured at 37 °C under 5% CO_2_ in an incubator. The Hela and A549 cells were resuspended to a concentration of 1–5 × 10^4^ cell/mL in Dulbecco’s modified Eagle’s medium (DMEM, Gibco). The Spca-1 cell line were maintained in Roswell Park Memorial Institute 1640 medium (RPMI1640, Biowest) supplemented with 10% foetal bovine serum (FBS), 100 U/mL penicillin, and 100 μg/mL streptomycin. The effects on growth inhibition were evaluated by MTT method. Aliquots of 100 μL cell suspension and 100 μL rLZ-8/rFIP-fve/rFIP-bbo (2, 4, 8, 16, 32, and 64 μg/mL) were seeded into a 96-well microplate. PBS was used as negative control. After cultivation for 24 h at 37 °C in a humidified 5% CO_2_ incubator, the percentage of viable cells was measured by MTT assay. Absorbance (OD) was measured at 570 nm using a Multiskan MK3.

### Tumour cell apoptosis assay

To observe if rLZ-8/rFIP-fve/rFIP-bbo induced apoptosis, 800 mL tumour cell suspension (1 × 10^6^ cells/mL) and 200 μL rLZ-8/rFIP-fve/rFIP-bbo (8 μg/ml) in PBS were incubated in a 24-well microplate at 37 °C 5% CO2 for 24 h. Untreated cells were used as negative controls. The percentage of apoptosis was evaluated using the Annexin V-EGFP/PI Cell Apoptosis Detection Kit (KeyGEN BioTECH, Nanjing, China) and then analysed using a flow cytometer (BD Accri C6, San Jose, CA, USA) with Cell-Quest software.

When cells die, chromatin contracted. Cover glasses were put in 6-well plates. Cells were inoculated into the plates at 5 × 10^5^ cells/well and incubated for 24 h. Into the cells 8 μg/mL of LZ-8, rFIP-fve and rFIP-bbo, was added and incubated for 24 h. The cell apoptosis staining was evaluated using the Hoechst Staining Kit (Beyotime, Shanghai, China), according to the manufacturer’s protocol. Staining was analysed using laser confocal fluorescence microscopy (Carl Zeiss Jena, Oberkochen, DER). The culture medium was used as a negative control.

### Migration inhibition assay

The wound-healing assay was used to compare the inhibitory effect of rLZ-8, rFIP-fve, and rFIP-bbo on the migration of the cells. The cells were cultured on 6-well plates and grown in a medium containing 10% FBS to a nearly confluent cell monolayer. A plastic pipette tip was used to draw a linear “wound” on the cell monolayer of each well. The monolayer was subsequently washed twice using PBS to remove debris or detached cells, and 8 μg/mL of rLZ-8, rFIP-fve and rFIP-bbo was added to the plates, respectively. PBS was added to the negative control. After 24 h and 36 h of incubation, the morphology of the cells was observed using an inverted microscope (Shanghai Cai Kang Optical Instrument Co., LTD, Shanghai, China), and captured by a digital camera coupled to the microscope (magnification, 200×).

### Statistical analysis

All values presented are mean ± standard deviation (SD). Each experiment was repeated thrice. Statistical comparisons between treatments were calculated by unpaired t-tests using GraphPad Prism 5 Software (GraphPad Software Inc., La Jolla, CA, USA). The values were significant when the p value was smaller than 0.05 between the two treatments.

## Supplementary information

Supplementary Information

## References

[1] Li QZ, Wang XF, Zhou XW (2011). Recent status and prospects of the fungal immunomodulatory protein family. Crit. Rev. Biotechnol..

[2] Paaventhan P (2003). A 1.7 Å structure of Fve, a member of the new fungal immunomodulatory protein family. J. Mol. Biol..

[3] Huang L (2009). Crystal structure of LZ-8 from the medicinal fungus *Ganoderma lucidium*. Proteins Struct. Funct. Bioinf..

[4] Uribe-Echeverry PT, Lopez-Gartner GA (2017). Fungal immunomodulatory proteins in the context of biomedicine. Front. Biosci..

[5] Lg VDH, Ja VDV, Kino K, Hoitsma AJ, Tax WJ (1996). Ling-Zhi-8: a fungal protein with immunomodulatory effects. Transplant. Proc..

[6] Xu H (2016). Recombinant FIP-gat, a fungal immunomodulatory protein from *Ganoderma atrum* induces growth inhibition and cell death in breast cancer cells. J. Agr. Food Chem..

[7] Lin TY, Hsu HY, Sun WH, Wu TH, Tsao SM (2017). Induction of Cbl-dependent epidermal growth factor receptor degradation in Ling Zhi-8 suppressed lung cancer. Int. J. Cancer.

[8] Chang YC (2013). Interruption of lung cancer cell migration and proliferation by fungal immunomodulatory protein FIP-fve from *Flammulina velutipes*. J. Agr. Food Chem..

[9] Wu CT (2011). Ling Zhi-8 mediates p53-dependent growth arrest of lung cancer cells proliferation via the ribosomal protein S7-MDM2-p53 pathway. Carcinogenesis.

[10] Li S (2016). Identification and Characterisation of a Novel Protein FIP-sch3 from *Stachybotrys chartarum*. PloS one.

[11] Li S, Nie Y, Ding Y, Shi L, Tang X (2014). Recombinant expression of a novel fungal immunomodulatory protein with human tumor cell antiproliferative activity from *Nectria haematococca*. Int. J. Mol. Sci..

[12] Li S, Shi L, Ding Y, Nie Y, Tang X (2015). Identification and functional characterization of a novel fungal immunomodulatory protein from *Postia placenta*. Food Chem. Toxicol..

[13] Li S (2017). FIP-sch2, a new fungal immunomodulatory protein from S*tachybotrys chlorohalonata*, suppresses proliferation and migration in lung cancer cells. Appl. Microbiol. Biot..

[14] Pushparajah V (2016). Characterisation of a new fungal immunomodulatory protein from tiger milk mushroom, *Lignosus rhinocerotis*. Sci. Rep-UK..

[15] Li S (2017). Characterization of a new fungal immunomodulatory protein, FIP-dsq2 from *Dichomitus squalens*. J. Biotechnol..

[16] Wang HJ, Hsiao YY, Chen YP, Ma TY, Tseng CP (2016). Polarity alteration of calcium site induces a hydrophobic interaction network and enhances Cel9A endoglucanase thermostability. Appl. Environ. Microb..

[17] Lg VDH (1995). Ling Zhi-8: studies of a new immunomodulating agent. Transplantation.

[18] Huang WN, Yang CY, Chen DC, Chuang LT (2014). Correlation of the structure and bioactivity of recombinant fungal immunomodulatory protein, ling-zhi-8 (lz-8) following exposure to denaturing conditions. J. Food Biochem..

[19] Chu PY (2015). Oral fungal immunomodulatory protein-*Flammulina velutipes* has influence on pulmonary inflammatory process and potential treatment for allergic airway disease: A mouse model. J. Microbiol. Immunol..

[20] Riley R (2014). Extensive sampling of basidiomycete genomes demonstrates inadequacy of the white-rot/brown-rot paradigm for wood decay fungi. P. Natl. Acad. Sci. USA.

[21] Lin WH, Hung CH, Hsu CI, Lin JY (1997). Dimerization of the N-terminal amphipathic α-helix domain of the fungal immunomodulatory protein from *Ganoderma tsugae* (Fip-gts) defined by a yeast two-hybrid system and site-directed mutagenesis. J. Biol. Chem..

[22] Wang Y (2016). Discovery and characterization of the highly active fungal immunomodulatory protein Fip-vvo82. J. Chem. Inf. Model..

[23] Bao DP, Bai R, Gao YN, Wu YY, Wang Y (2018). Computational Insights into the molecular mechanism of the high immunomodulatory activity of lz-8 protein isolated from the Lingzhi or Reishi medicinal mushroom ganoderma lucidum (agaricomycetes). Int. J. Med. Mushrooms.

[24] Felsenstein J (1985). Confidence limits on phylogenies: an approach using the bootstrap. Evolution.

[25] Fiser A, Do RKG (2000). Modeling of loops in protein structures. Protein sci..

[26] Laskowski RA, MacArthur MW, Moss DS, Thornton JM (1993). PROCHECK: a program to check the stereochemical quality of protein structures. J. Appl. Crystallogr..

[27] Sander P (2013). GROMACS 4.5: a high-throughput and highly parallel open source molecular simulation toolkit. Bioinformatics.

[28] Nicolas S, D Peter T (2011). Combination of the CHARMM27 force field with united-atom lipid force fields. J. Comput. Chem..

[29] Delano WL (2002). The PyMol molecular graphics system. Proteins..

[30] Chen R, Li L, Weng Z (2003). ZDOCK: an initial-stage protein-docking algorithm. Proteins.

[31] Li L, Chen R, Weng Z (2003). RDOCK: Refinement of rigid‐body protein docking predictions. Proteins: Struct. Funct. Bioinf..

[32] Baker NA, Sept D, Joseph S, Holst MJ, Mccammon JA (2001). Electrostatics of nanosystems: application to microtubules and the ribosome. Proc. Natl. Acad. Sci. USA.

[33] Kumari R, Kumar R, Lynn A (2014). g_mmpbsa–a GROMACS tool for high-throughput MM-PBSA calculations. J. Chem. Inf. Model..

[34] Wang, Y. J. *et al.* Computational studies on the substrate interactions of influenza a virus PB2 subunit. *PLoS one.* **7**, e44079 (2012). 10.1371/journal.pone.0044079 PMC3434214 22957044

