# Supplementary material for: Identification of a Novel Anti-cancer Protein, FIP-bbo, from Botryobasidium botryosum and Protein Structure Analysis using Molecular Dynamic Simulation
Source: Sci Rep. 2019 Apr 9;9:5818. doi: 10.1038/s41598-019-42104-1 (PMC6456589; doi:10.1038/s41598-019-42104-1)
Supplement: Supplementary file 1 — Supplementary Information [file 41598_2019_42104_MOESM1_ESM.docx]

Supporting Information：

**Identification of a Novel Anti-cancer Protein, FIP-bbo, from** ***Botryobasidium botryosum* and Protein Structure Analysis using Molecular Dynamic Simulation**

**Y. Wang^1, +^, Y. N. Gao^1, +^, R. Bai^2, +^, H. Y. Chen^1^, Y. Y. Wu^1^, J. J. Shang^1^, & D. P. Bao^1*^**

^1^National Engineering Research Center of Edible Fungi, Key Laboratory of Applied Mycological Resources and Utilization, Ministry of Agriculture, Shanghai Key Laboratory of Agricultural Genetics and Breeding, Institute of Edible Fungi, Shanghai Academy of Agriculture Science, Shanghai, 201403, People’s Republic of China

^2^College of Food Science, Shanghai Ocean University, Shanghai, 201306, People’s Republic of China

Correspondence and requests for materials should be addressed to D*.* P*.* B. (email: baodp@hotmail.com )

^+^these authors contributed equally to this work


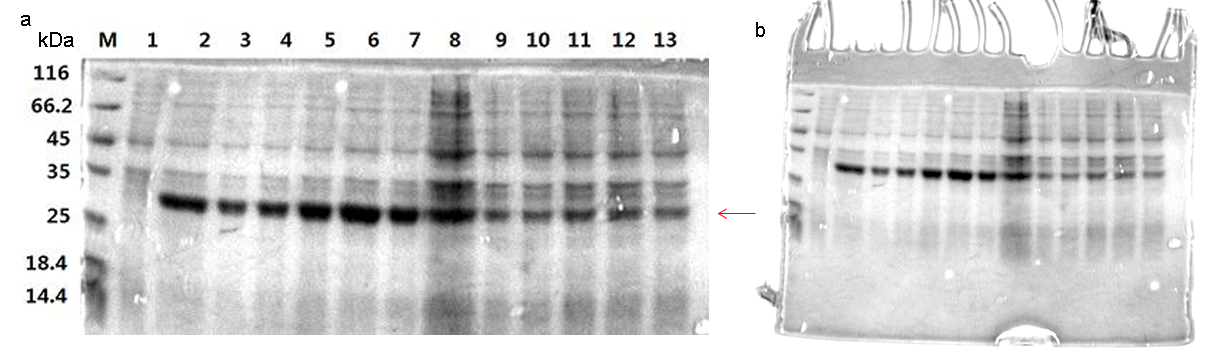


Figure S1. (a) Effect of final concentration of IPTG, induction time and culture temperature on expression of fusion protein SUMO-FIP-bbo. Lane 1: *E. coli* cultures without IPTG induction; Lanes 2-4: SUMO-FIP-bbo incubated with 1.0 mM IPTG in 37 °C for 4h; Lanes 5-7: SUMO-FIP-bbo incubated with 0.2 mM IPTG in 37 °C for 16h; Lanes 8-10: SUMO-FIP-bbo incubated with 1.0 mM IPTG in 15 °C for 4h; Lanes 11-13: SUMO-FIP-bbo incubated with 0.2 mM IPTG in 15 °C for 16h. M: broad range protein marker. The arrow mark indicated the target SUMO-FIP-bbo. (b) The full-length gel of this SDS-PAGE analysis.


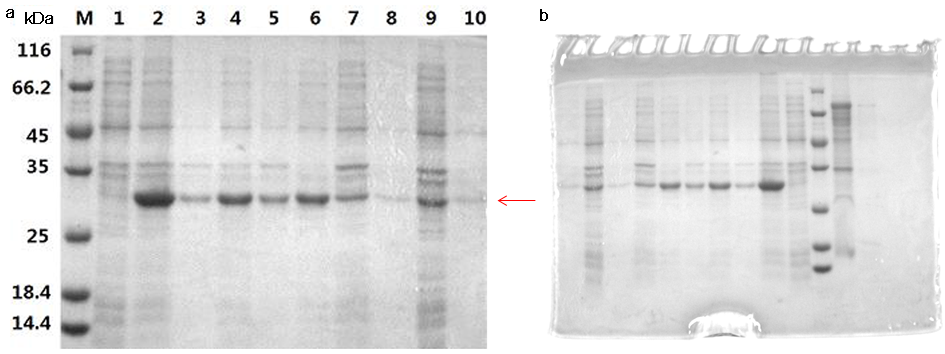


Figure S2. (a) SDS-PAGE analysis of the supernatant and precipitant fractions of the cell lysate. Lane M: broad range protein marker. Lane 1: *E. coli* cultures without IPTG induction; Lane 2: the fusion protein SUMO-FIP-bbo incubated with 1.0 mM IPTG in 37 °C; Lane 3: the precipitant fractions of the cell lysate (induction condition: 1.0 mM IPTG in 37 °C); Lane 4: the supernatant fractions of the cell lysate (induction condition: 1.0 mM IPTG in 37 °C); Lane 5: the precipitant fractions of the cell lysate (induction condition: 0.2 mM IPTG in 37 °C); Lane 6: the supernatant fractions of the cell lysate (induction condition: 0.2 mM IPTG in 37 °C); Lane 7: the precipitant fractions of the cell lysate (induction condition: 1.0 mM IPTG in 15 °C); Lane 8: the supernatant fractions of the cell lysate (induction condition: 1.0 mM IPTG in 15 °C); Lane 9: the precipitant fractions of the cell lysate (induction condition: 0.2 mM IPTG in 15 °C); Lane 10: the supernatant fractions of the cell lysate (induction condition: 0.2 mM IPTG in 15 °C). The arrow mark indicated the target SUMO-FIP-bbo. (b) The full-length gel of this SDS-PAGE analysis.


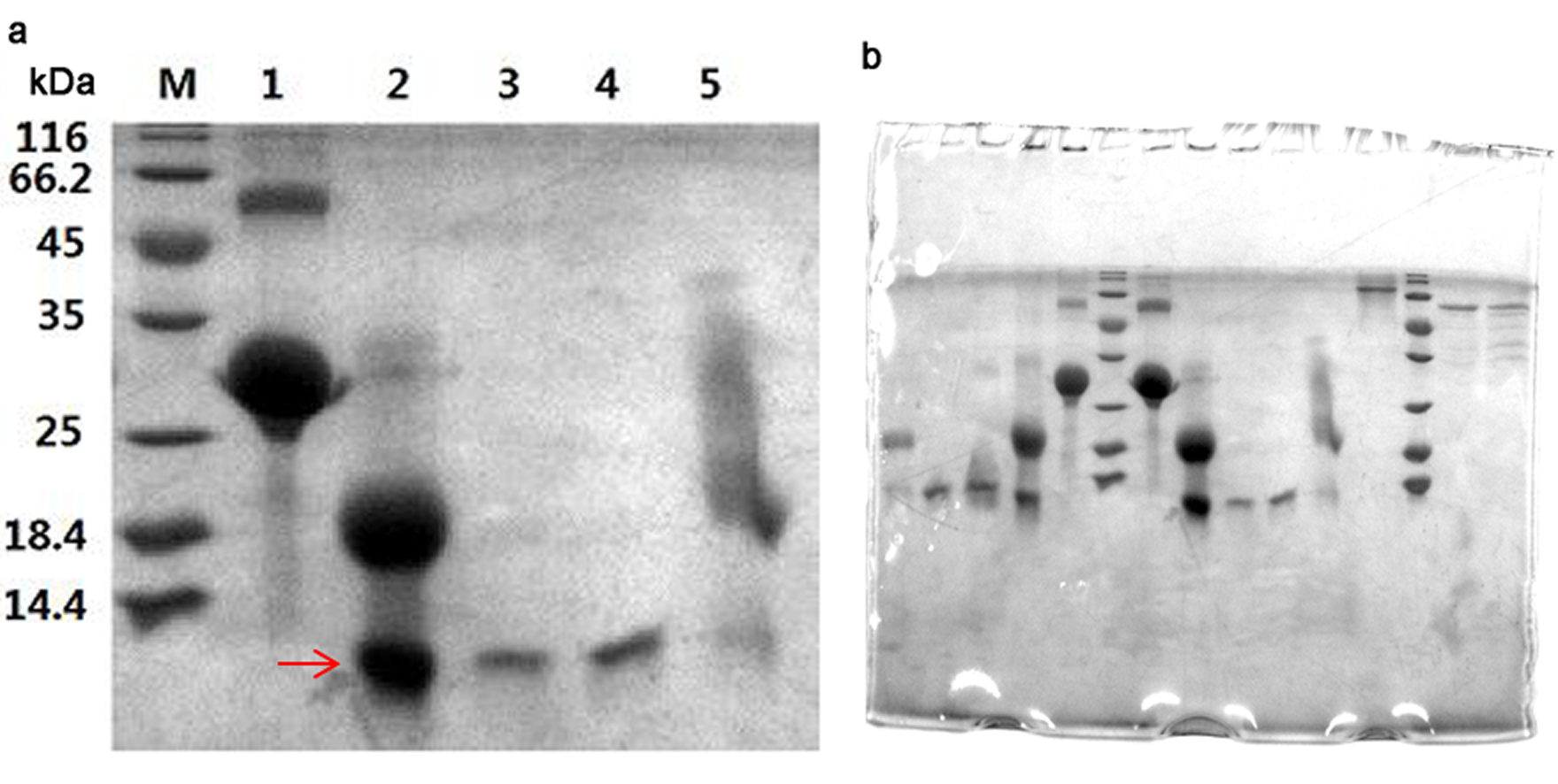


Figure S3 (a) Cleavage of SUMO-tag of fusion protein SUMO-FIP-bbo. Lane M: Broad range protein marker. Lane 1: sample before SUMO protease cleavage. Lane 2: sample after SUMO protease cleavage. Lane 3, 4: target FIP-bbo purified by Ni-NTA agarose. Lane 5: the eluent eluted by buffer (20mM Tris, 300mM NaCl, 10% Glycerol, 250mM imidazole, pH8.0). The arrow mark indicated the target FIP-bbo. (b) The full-length gel of this SDS-PAGE analysis.


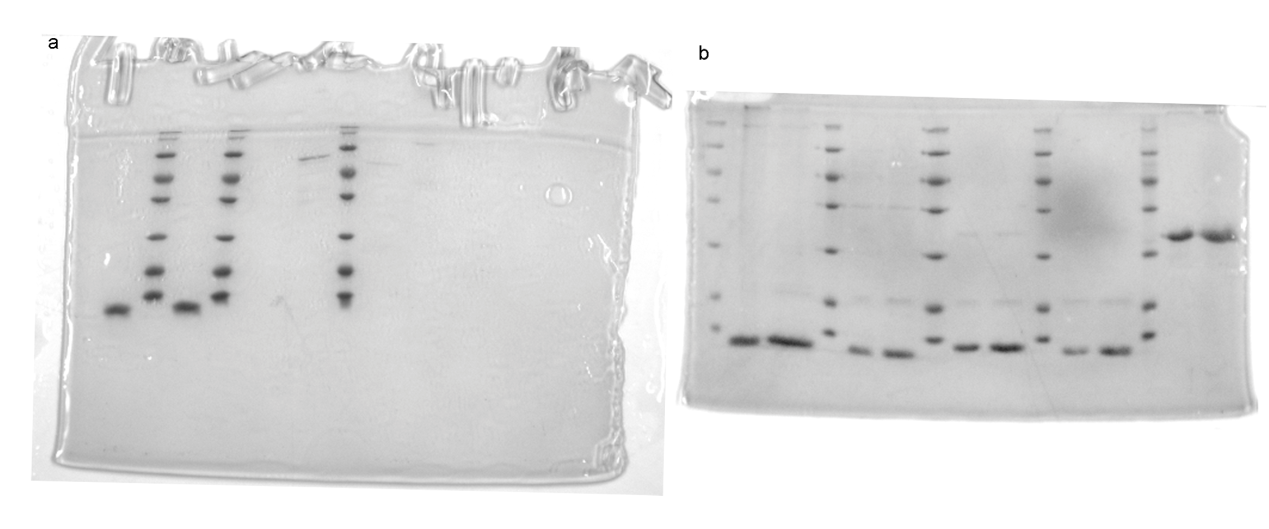


Figure S4.The full-length gel of SDS-PAGE analysis of rFIP-fve (a), rFIP-bbo (a) and rLZ-8 (b).
